# Nanohydroxyapatite-reinforced chitosan composite hydrogel for bone tissue repair in vitro and in vivo

**DOI:** 10.1186/s12951-015-0099-z

**Published:** 2015-06-12

**Authors:** S Dhivya, S Saravanan, T P Sastry, N Selvamurugan

**Affiliations:** Department of Biotechnology, School of Bioengineering, SRM University, Kattankulathur, 603203 Tamil Nadu India; Bioproducts Laboratory, Central Leather Research Institute, Chennai, 600 020 Tamil Nadu India

**Keywords:** Chitosan, Zinc, nHAp, β-Glycerophosphate, Runx2, Bone

## Abstract

**Background:**

Bone loss during trauma, surgeries, and tumor resection often results in critical-sized bone defects that need to be filled with substitutionary materials. Complications associated with conventional grafting techniques have led to the development of bioactive tissue-engineered bone scaffolds. The potential application of hydrogels as three-dimensional (3D) matrices in tissue engineering has gained attention in recent years because of the superior sensitivity, injectability, and minimal invasive properties of hydrogels. Improvements in the bioactivity and mechanical strength of hydrogels can be achieved with the addition of ceramics. Based on the features required for bone regeneration, an injectable thermosensitive hydrogel containing zinc-doped chitosan/nanohydroxyapatite/beta-glycerophosphate (Zn-CS/nHAp/β-GP) was prepared and characterized, and the effect of nHAp on the hydrogel was examined.

**Methods:**

Hydrogels (Zn-CS/β-GP, Zn-CS/nHAp/β-GP) were prepared using the sol–gel method. Characterization was carried out by scanning electron microscopy (SEM), energy dispersive spectroscopy (EDX), Fourier transform infrared spectroscopy (FTIR), and X-ray diffraction (XRD) as well as swelling, protein adsorption, and exogenous biomineralization studies. Expression of osteoblast marker genes was determined by real-time reverse transcriptase polymerase chain reaction (RT-PCR) and western blot analyses. In vivo bone formation was studied using a rat bone defect model system.

**Results:**

The hydrogels exhibited sol–gel transition at 37°C. The presence of nHAp in the Zn-CS/nHAp/β-GP hydrogel enhanced swelling, protein adsorption, and exogenous biomineralization. The hydrogel was found to be non-toxic to mesenchymal stem cells. The addition of nHAp to the hydrogel also enhanced osteoblast differentiation under osteogenic conditions in vitro and accelerated bone formation in vivo as seen from the depositions of apatite and collagen.

**Conclusions:**

The synthesized injectable hydrogel (Zn-CS/nHAp/β-GP) showed its potential toward bone formation at molecular and cellular levels in vitro and in vivo. The current findings demonstrate the importance of adding nHAp to the hydrogel, thereby accelerating potential clinical application toward bone regeneration.

## Background

The rapid shift in the treatment regime from bone grafting to bone tissue engineering is due to surgical complications and biocompatibility issues in terms of immunogenic responses associated with tissue grafting [1, 2]. The use of polymeric scaffolds and ceramic particles has shown promising improvements, extending their application as bone graft materials. Hydrogels have mechanical and structural properties that are very similar to those of natural tissues and extracellular matrices (ECMs) [3]. Hydrogels are exemplified as bone grafts in non-load-bearing regions because of their high degree of flexibility, low toxicity, biocompatibility, biodegradability, sensitivity to external stimuli, injectability, and easily modifiable properties and because they can be used for timed release of growth factors [4–8].

Chitosan (CS), which is a deacetylated derivative of chitin, is a linear aminopolysaccharide that has been widely used in biomedical applications [9–14]. CS possesses both structural and compositional similarity to glycosaminoglycans (GAGs) and hence elicits minimal immune response [15]. It demonstrates efficient chelating abilities with metal ions [16] and exhibits swelling properties [17]. CS-based hydrogels have the potential to swell and dehydrate depending on the surrounding environment [18]. Zinc (Zn) is an important trace element found in bone and has structural and regulatory cellular functions [19]. It stimulates osteoblastogenesis and suppresses osteoclastogenesis [20, 21]. In addition, Zn possesses excellent antimicrobial properties [22], which are widely exploited in bone implant materials to tackle implant-associated microbial infections.

Hydroxyapatite (HAp) is a mineral component of natural bone and exhibits osteoconductive properties, osteoinductive properties, bone bonding abilities, and slow degradation in situ [23]. A significant increase in protein adsorption and osteoblast adhesion was observed on nanosized ceramic materials, compared with that on traditional micron-sized ceramic materials [24]. The addition of HAp improves stiffness, interconnectivity, and osteogenic potential in collagen-based scaffolds for bone tissue engineering applications [25]. Organic phosphates, in particular β-glycerophosphate (β-GP), have been used to induce mineralization in cell culture systems [26]. β-GP serves as a source of inorganic phosphates when hydrolyzed by alkaline phosphatase (ALP), and it has been used as an osteogenic supplement in cultures of human bone marrow stromal cells (hBMSCs) [27, 28]. By adding β-GP, which is a weak base, to aqueous chitosan solutions, the polymer remains in solution at neutral pH and room temperature, while homogeneous gelation of this system can be triggered by heat [29]. Controlled hydrogel formation with temperature increase takes place because of the thermally induced transfer of protons from the amine groups of chitosan to the (PO_4_)^2−^ groups of β-GP [30, 31].

Based on the biomaterial properties mentioned above, in this study, we aimed to synthesize and characterize an injectable thermosensitive hydrogel containing zinc-doped chitosan/nanohydroxyapatite/β-glycerophosphate (Zn-CS/nHAp/β-GP). Most importantly, the impact of nHAp on the osteogenic potential of the synthesized hydrogel was determined in vitro and in vivo. The molecular mechanism behind the bone formation potential of the hydrogel was also examined. In vitro bone regeneration promoted by the hydrogel was confirmed in vivo in critical-sized bone defects in rats.

## Results and discussion

### Hydrogel preparation

Zn-CS/β-GP and Zn-CS/nHAp/β-GP solutions were prepared by drop-wise addition of an optimized molar concentration of pre-cooled β-GP to pre-cooled solutions of Zn-CS and Zn-CS/nHAp under continuous mixing. The sol form of the hydrogel formed at 4°C was transferred to 37°C for gelation. The gelation time was found to be >10 min at pH 7. The acidic pH of the CS solution changed to 7 with the addition of β-GP.

### Physicochemical characterization of hydrogel

**Figure 1:**
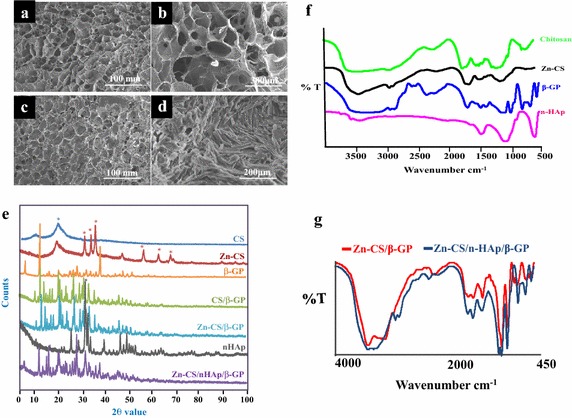
Physicochemical characterization of the hydrogels. Scanning electron microscopic images of **a** Zn-CS/β-GP and **c** Zn-CS/nHAp/β-GP; **b** and **d** are magnified images of the above, respectively. Pores greater than 150 μm in diameter were seen in both hydrogels. The presence of nHAp along the matrix of chitosan is clearly visible under high magnification. **e** XRD spectra of the individual components and of the hydrogels. FTIR spectra of **f** the individual components and of **g** the Zn-CS/β-GP and Zn-CS/nHAp/β-GP hydrogels.

Scanning electron microscopy (SEM) analysis of the Zn-CS/β-GP hydrogel (Figure 1a, b) and the Zn-CS/nHAp/β-GP hydrogel (Figure 1c, d) revealed porous architectures indicative of the presence of both micro- and macro-sized pores. The pores in the hydrogels were uniformly present and highly interconnected. In both Zn-CS/β-GP and Zn-CS/nHAp/β-GP hydrogels, the diameter of the pores was in the range of 100–150 µm. This was a suitable pore size for cell penetration into the hydrogels and aided in nutrient transport and metabolic waste disposal. The magnified image of the hydrogel with nHAp shown in Figure 1d indicates that the addition of nHAp resulted in the deposition of sharp needle-shaped nanoparticles along the pore walls and struts, which acted as nucleating sites for mineral deposition, protein adsorption, and cell attachment because of their nanoarchitecture [32–34].

X-ray diffraction (XRD) analyses of the individual components employed in the preparation of the hydrogels and the final resulting hydrogels are shown in Figure 1e. The appearance of broad peaks between 20° and 30° indicated the semi-crystalline nature of chitosan. The sharper peaks observed at 31.66°, 34.2°, 36.16°, 47.86°, 56.52°, 62.66°, and 67.80° corresponded to the (100), (002), (101), (102), (110), (103), and (112) lattice planes, respectively, of the wurtzite phase of ZnO. The values are in strong agreement with those of standard ZnO in JCPDS card No 36-1451 [35]. The occurrence of phases corresponding to CS, ZnO, nHAp, and β-GP was clearly evident in the final hydrogel because of its crystalline nature.

The Fourier transform infrared spectroscopy (FTIR) spectra of the individual components and of the hydrogels are shown in Figure 1f and g. The absorption band at 1,600 cm^−1^, representing the “free amine” group of chitosan, disappeared in the Zn-CS complex and was replaced by a new peak at 1,657 cm^−1^, corresponding to the Zn-CS complex [36]. Chitosan showed an absorption peak at 3,425 cm^−1^, which was attributed to the combined peaks of the –NH_2_ and –OH stretching vibration. This broader and stronger peak of chitosan was shifted to a higher wavenumber at 3,430 cm^−1^ in Zn-CS, indicating a strong interaction between these groups and ZnO. The absorption peak at 2,921 cm^−1^ was attributed to the asymmetric stretching of–CH_3_ in chitosan, and the absorption peaks at 1,657 and 1,093 cm^−1^ were ascribed to the bending vibration of the –NH_2_ group and the C–O stretching group, respectively. All of these interactions clearly described the attachment of zinc oxide to chitosan chains. Standard FTIR spectra of nHAp and β-GP were also recorded to study the interactions among the gel-forming components. The spectrum of β-GP showed the bending and stretching vibration modes of PO_4_, which were identified by the peaks at 548 and 653 cm^−1^, respectively. The individual spectrum of nHAp showed the ϒ_1_ and ϒ_4_ absorption bands of PO_4_ at 1,050 and 577 cm^−1^, respectively [37]. In addition, the peak at 3,567 cm^−1^ in the nHAp spectrum corresponded to the –OH stretching mode of stoichiometric HAp [38].

The FTIR spectrum of the Zn-CS/β-GP hydrogel was compared with that of the Zn-CS/nHAp/β-GP hydrogel (Figure 1g) to identify important interactions between Zn-CS/β-GP and the bioactive component nHAp. The Zn-CS/nHAp/β-GP hydrogel revealed peaks at 1,410 and 1,573 cm^−1^ attributing to C=O stretching, strong peaks at 2,848 and 2,936 cm^−1^ belonging to the C–H stretching region, and small peaks between 2,160 and 2,255 cm^−1^ corresponding to C=C. The peaks at 1,074 and 2,848 cm^−1^ indicated amide II bands, and the peaks at 1,664, 1,573, and 1,550 cm^−1^ were assigned to amide I (C=O), amino (–NH_2_), and amide II (–NH), respectively [39]. The peak at 960 cm^−1^ in the Zn-CS/β-GP hydrogel, representing the ϒ_1_ absorption band of PO_4_, was intensified and shifted to a higher wavenumber of 971 cm^−1^ with the addition of nHAp. This result indicated a strong interaction between nHAp and Zn-CS in the hydrogel.

### Hydrogel characteristics with the addition of nHAp

**Figure 2:**
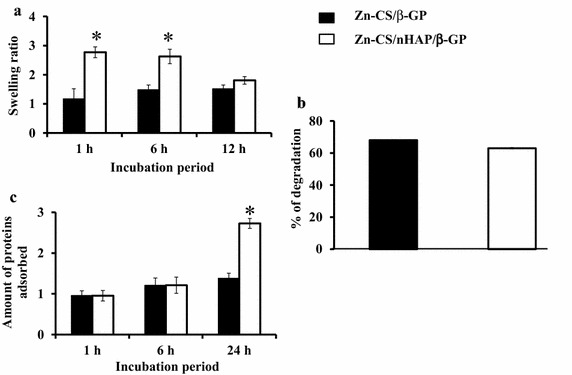
**a** Swelling studies of Zn-CS/β-GP and Zn-CS/nHAp/β-GP hydrogels. Addition of nHAp significantly increased the water retention capacity of the hydrogel. **b** Degradation studies of Zn-CS/β-GP and Zn-CS/nHAp/β-GP hydrogels. Addition of nHAP had no effect on the degradation characteristics of the hydrogel. *Asterisk* indicates significant changes compared with Zn-CS/β-GP (*p* < 0.05) (n = 6). **c** Protein adsorption studies on Zn-CS/β-GP and Zn-CS/nHAp/β-GP hydrogels.

Swelling behavior and structural integrity are two critical design variables in constructing hydrogels and other scaffolding materials. CS has the ability to readily swell up when exposed in a biological environment. Figure 2a indicates increased fluid uptake associated with the addition of nHAp particles to the hydrogel. A drop in the swelling behavior was noticed with an increase in the incubation period to greater than 12 h. The higher rate of swelling associated with hydrogels containing nHAp may be due to the hydrophilicity offered by the free –OH groups of nHAp. Fluid retention results in the relaxation of mechanically coiled chains of chitosan, leading to an increase in surface area. In turn, the increase in internal surface area caused by swelling facilitates the infiltration of cells into the scaffolds. Moreover, increased swelling enables efficient transport of nutrients from the culture media and ensures better probability of cell infusion in a 3D pattern, mimicking cell growth under physiological conditions in vivo [40, 41]. The persistence and rate of degradation of any implanted material in vivo are the key factors that affect the rate of bone formation. Glycosidic bonds in the CS polymeric network are a major target for hydrolytic scission by lysozymes. The chains of *N*-acetylglucosamine in CS are linked by glycosidic bonds, and their degradation will lead to the release of amino sugars, which can be utilized for several metabolic pathways [42]. In addition, the degradation of scaffolds is essential for the release of bioactive ingredients and determines the mechanical strength of any biomaterial. The degradation rate of both hydrogels remained the same at 48 h. From Figure 2b, it is clear that the addition of nHAp had no effect on the degradation of the hydrogel.

Protein adsorption is a pre-requisite for cell-biomaterial interaction, which can be strengthened by the presence of nanoparticles in the composites. The protein adsorption ability of the hydrogels was studied at different times (Figure 2c). Initial protein adsorption is a vital property of a successful tissue-engineered implant. The addition of nHAp significantly increased the amount of protein adsorbed onto the hydrogel at 24 h. This effect could have been due to the increased hydrophilicity of the hydrogel, which mediated enhanced protein adsorption. In addition, the nanostructures of hydroxyapatite in the hydrogel offered increased surface area for the adsorption of proteins [43].

**Figure 3:**
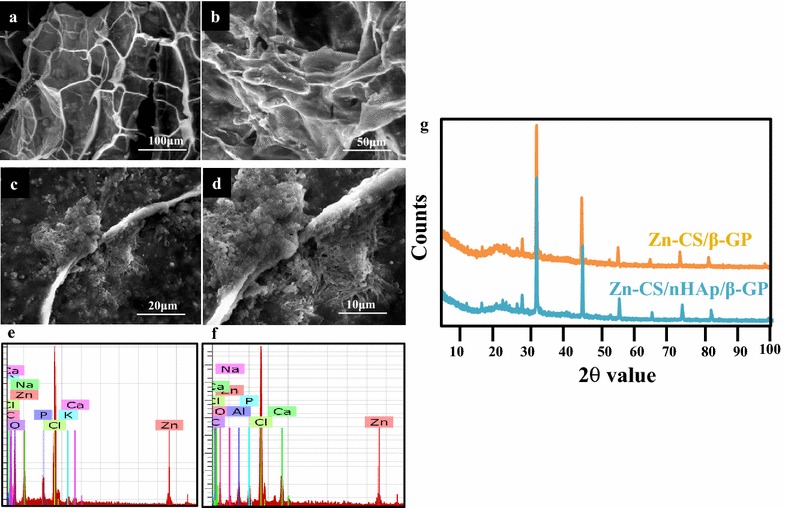
SEM images of exogenous biomineralized **a** Zn-CS/β-GP and **b** Zn-CS/nHAp/β-GP hydrogels in SBF for 21 days. **c** and **d** are *magnified images* of the above, respectively. EDX spectra of **e** Zn-CS/β-GP and **f** Zn-CS/nHAp/β-GP hydrogels. **g** XRD spectra of the mineralized hydrogels.

The in vitro exogenous biomineralization of the hydrogels prepared was examined by immersing them in simulated body fluid (SBF) solutions for 21 days. The presence of apatite crystals on the surface of the hydrogels was examined and confirmed by SEM analyses (Figure 3a–d). Figure 3a and b represent the SEM images of exogenous biomineralized Zn-CS/β-GP and Zn-CS/nHAp/β-GP hydrogels, respectively. Figure 3c and d represent the magnified images of the above. The pattern of apatite formation was intense on the surface of the hydrogels containing nHAp (Figure 3d). This could be due to the availability of negatively charged hydroxyl groups on hydroxyapatite that acted as nucleation sites to initiate crystal deposition. Furthermore, the presence of calcium and phosphate ions in the biomineralized Zn-CS/β-GP (Figure 3e) and Zn-CS/nHAp/β-GP (Figure 3f) hydrogels was confirmed in the energy dispersive spectroscopy (EDX) spectra. The characteristic diffraction peaks of hydroxyapatite at values of 2θ from 31.8° to 52.5°, in accordance to JCPDS-09-0432, were observed in all tested samples (Figure 3g). The observed sharper peaks implied that the degree of crystallinity of the hydrogel samples had increased after biomineralization.

The antimicrobial nature of any implanted graft material aids in the persistence of the implant by preventing microbial attacks associated with surgeries. The hydrogels exhibited a bactericidal effect against Gram-negative and Gram-positive bacteria, such as *Escherichia coli* and *Streptococcus pyogenes*, respectively. The antimicrobial activity of the Zn-CS/nHAp/β-GP hydrogel was not significantly different compared with that of the Zn-CS/β-GP hydrogel (Table 1). Overall, these results (Figures 2, 3) clearly indicated the superiority of the nHAp-incorporated hydrogel over the native hydrogel (without nHAp).

**Table 1:** Zone of inhibition observed against Gram-negative and Gram-positive organisms

Hydrogel	Zone of inhibition (mm)	
	*Escherichia* *coli*	*Streptococcus* *pyogenes*
Zn-CS/β-GP	8.0 ± 0.013	9.3 ± 0.305
Zn-CS/n-HAp/β-GP	8.0 ± 0.057	9.6 ± 0.252

Addition of nHAp induced no significant change in the antimicrobial behavior of the hydrogel.

### Biocompatible nature of the synthesized hydrogel

**Figure 4:**
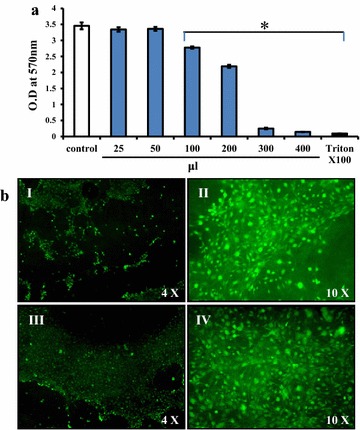
In vitro cytotoxicity analysis of Zn-CS/nHAp/β-GP hydrogel. **a** MTT assay of C3H10T1/2 cells grown for 24 h in medium conditioned with Zn-CS/nHAp/β-GP hydrogel. Wells treated with 0.1% Triton X-100 served as positive controls. *Asterisk* indicates significant decrease compared with control (p < 0.05) (n = 5). **b** FDA staining of C3H10T1/2 cells grown in Zn-CS/nHAp/β-GP hydrogel for 5 days and assessed with ×4 and ×10 objectives for cellular morphology. Control cells (*I*) and cells grown in hydrogel (*III*) showed highly spread cellular extensions. *Magnified images* of control and hydrogel-treated cells are depicted in (*II*) and (*IV*), respectively.

Biomaterials intended for tissue engineering applications should not exert any toxicity on mammalian cells. The dissolution of ions from biomaterials plays a vital role in regulating cytotoxicity. Impedance of cell growth and alterations in membrane permeability are signs of cytotoxicity. Hence, the synthesized Zn-CS/nHAp/β-GP hydrogel was subjected to an indirect 3-(4,5-dimethythiazol-2-yl)-2,5-diphenyltetrazolium bromide (MTT) assay. Mouse mesenchymal stem cells (mMSCs) were incubated with medium that was conditioned by immersing hydrogels therein for 24 h. Cells incubated in normal media served as negative controls, and cells treated with 0.1% Triton X-100 served as positive controls. From Figure 4a, it is obvious that the cells experienced cytotoxicity when cultured in 100 μL of conditioned medium, which was the amount equivalent to 5 mg of hydrogel. Since there was no discernible toxicity associated with 50 μL (2.5 mg) of conditioned medium, this condition was chosen for further studies. Direct measurement of cell viability was also performed with fluorescein diacetate (FDA) staining of control mMSCs (Figure 4bI, II) and of the mMSCs in the Zn-CS/nHAp/β-GP hydrogel (Figure 4bIII, IV). The results showed the non-toxic nature of the hydrogel, as indicated by the distinct cellular morphology and highly spread cellular extensions.

### mMSC differentiation to osteoblasts at the cellular level

**Figure 5:**
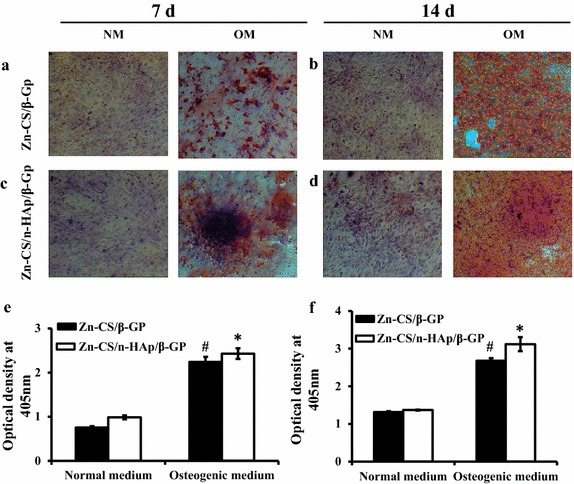
Effect of Zn-CS/nHAp/β-GP hydrogel on mMSC differentiation and mineralization by alizarin staining. Cells were grown in the presence of hydrogels under normal and osteogenic conditions for periods of 7 and 14 days. Fresh medium was replenished every 3 days. Cells were fixed and stained with alizarin red to visualize the calcium deposits. Representative microscopic images of the fixed cells incubated on **a** Zn-CS/β-GP hydrogel for 7 days, **b** Zn-CS/β-GP hydrogel for 14 days, **c** Zn-CS/nHAp/β-GP hydrogel for 7 days, and **d** Zn-CS/nHAp/β-GP hydrogel for 14 days. **e** and **f** are quantification plots of calcium deposits of the above. *NM* normal medium, *OM* osteogenic medium. *Asterisk* indicates significant increase compared with the respective control in normal medium. *Hash* indicates significant increase compared with the respective control in normal medium (*p* < 0.05).

To determine the capability of hydrogels and the significance of nHAp in promoting MSC differentiation into osteoblasts, mMSCs were grown in the conditioned media obtained from the Zn-CS/β-GP hydrogel or from the Zn-CS/nHAp/β-GP hydrogel, either in the presence or absence of osteogenic stimulants for 7 and 14 days (Figure 5). Calcium deposition was assessed by alizarin red staining. Microscopic images of the biomineralized deposits indicated that, in the presence of osteogenic stimulation, the addition of nHAp in the Zn-CS/β-GP hydrogel promoted better osteoblast differentiation at 7 days (Figure 5c) and 14 days (Figure 5d) compared with the Zn-CS/β-GP hydrogel (Figure 5a, b). Quantification data for the alizarin red staining also indicated increased mineralization in the Zn-CS/nHAp/β-GP hydrogel at 7 days (Figure 5e) and 14 days (Figure 5f). Hence, the hydrogel exerted maximal differentiation potential under osteogenic conditions and in the presence of nHAp, indicating its osteoconductive nature.

### mMSC differentiation into osteoblasts at the molecular level

**Figure 6:**
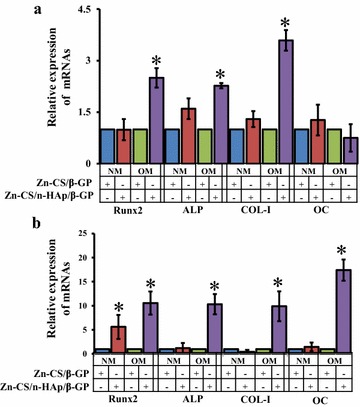
Effect of Zn-CS/nHAp/β-GP hydrogel on the mRNA expression of osteoblast differentiation marker genes in mMSCs. Cells were grown in the presence of Zn-CS/β-GP hydrogel or Zn-CS/nHAp/β-GP hydrogel under normal and osteogenic conditions for **a** 7 days and **b** 14 days. At the end of the incubation periods, total RNA was isolated and real-time RT-PCR was performed using primers specific for Runx2, ALP, COL-I, and OC genes. The fold change in mRNA expression was calculated over the control samples. GAPDH was used as an internal control. *NM* normal medium, *OM* osteogenic medium. *Asterisk* indicates significant increase compared with the respective control, i.e., Zn-CS/β-GP (*p* < 0.05).

Real-time reverse transcriptase polymerase chain reaction (RT-PCR) was carried out to determine the expression of osteoblastic differentiation marker genes such as alkaline phosphatase (ALP), type-I collagen (COL-I), osteocalcin (OC), and bone transcription factor Runx2 at the molecular level in mMSCs incubated with the hydrogels (Figure 6). Increased mRNA levels of ALP, COL-I, and Runx2 were observed in cells incubated with the hydrogel containing nHAp (Zn-CS/nHAp/β-GP) for 7 days in osteogenic medium compared with those observed in the Zn-CS/β-GP hydrogel (Figure 6a). A significant increase in the mRNA level of OC was found in mMSCs after 14 days of incubation with the Zn-CS/nHAp/β-GP hydrogel in the presence of osteogenic medium (Figure 6b). Although the mRNA expression of Runx2 was stimulated by nHAp in the Zn-CS/nHAp/β-GP hydrogel even in normal medium, maximal Runx2 expression was observed in osteogenic medium. Runx2 is an essential transcription factor for the expression of various osteoblast-specific marker genes, namely, ALP, COL-I, and OC [44]. ALP and COL-1 are early differentiation marker genes, whereas OC is a late marker gene of osteoblast differentiation. ALP plays a role in the conversion of inorganic pyrophosphate into inorganic phosphate, a process that enhances mineralization process [45]. COL-I aids in the deposition of collagen, the most abundant ECM protein in bone. The voids in the collagen molecules act as the sites of deposition for hydroxyapatite crystals, facilitating subsequent bone mineralization [46]. OC is a major non-collagenous protein component of the bone ECM, acting as a regulator of bone crystal growth by mediating its high affinity binding to hydroxyapatite crystals [47].

**Figure 7:**
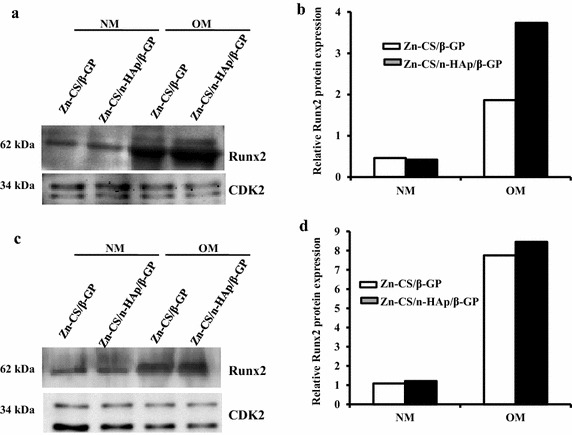
Effect of Zn-CS/nHAp/β-GP hydrogel on the expression of Runx2 protein. mMSCs were grown in the presence of Zn-CS/β-GP hydrogel or Zn-CS/nHAp/β-GP hydrogel under normal and osteogenic conditions for **a** 7 days and **c** 14 days. At the end of the incubation periods, whole cells lysates were prepared and separated onto 12% SDS-PAGE. The proteins were electrophoretically transferred to PVDF membranes and subjected to western blot analysis using Runx2 antibodies. Cdk2 antibodies were used as an internal loading control. **b** and **d** represent the quantification of Runx2 protein blots as mentioned above using Image Lab software version 4.1 (Bio-Rad). *NM* normal medium, *OM* osteogenic medium.

The expression of the Runx2 protein was also determined. At 7 and 14 days of cell incubation with Zn-CS/β-GP and Zn-CS/nHAp/β-GP hydrogels in osteogenic medium, the expression of Runx2 was higher than that in normal medium (Figure 7a, c). The addition of nHAp in the Zn-CS/β-GP hydrogel stimulated higher Runx2 protein expression than that seen in the Zn-CS/β-GP hydrogel at an incubation period of 7 days (Figure 7a). The densitometry scanning of western blotting is shown in Figure 7b and d. The consolidation of gene and protein expression results (Figures 6, 7) suggested that the presence of nHAp in the hydrogel enhanced the expression of Runx2 and osteoblast differentiation marker genes under osteogenic conditions, depicting the osteoconductive nature of the hydrogel.

### Healing of critical-sized bone defects in rat tibia in vivo

**Figure 8:**
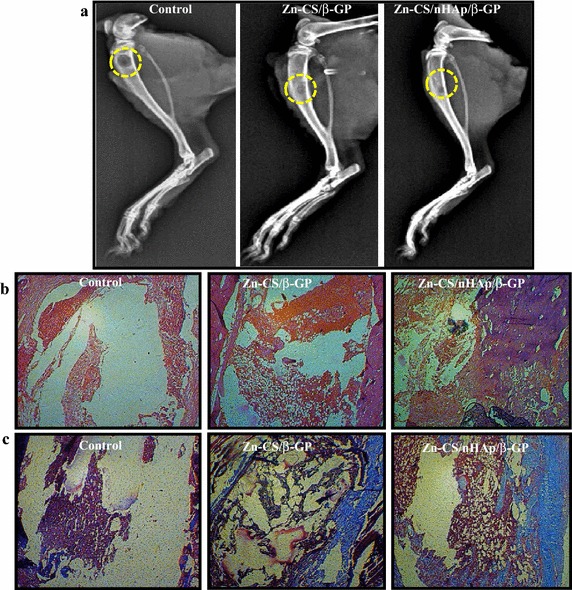
Effect of Zn-CS/nHAp/β-GP hydrogel on bone healing in vivo. **a** Radiographic images of the rat tibial defects obtained 2 weeks after they were filled with Zn-CS/β-GP hydrogel or Zn-CS/nHAp/β-GP hydrogel. **b** and **c** represent hematoxylin and eosin staining and collagen staining of the implants, respectively. Closure of the drill hole, formation of new bone, and deposition of collagen were seen in Zn-CS/nHAp/β-GP-treated animals. *Dotted yellow circle* indicates the drill hole defect and subsequent closure in hydrogel-treated animals. *Purple color-stained areas* indicate the formation of bone, and *blue-colored areas* depict the deposited collagen.

We next assessed the bone-healing property of the hydrogels in rats with tibial defects as well as the significance of nHAp in the hydrogels. From radiographic images taken 2 weeks post-surgery, better tissue organization was observed in tibiae treated with Zn-CS/nHAp/β-Gp hydrogels than in tibiae treated with Zn-CS/β-GP hydrogels and in control tibiae (Figure 8a). The radiographs showed that, in the control group, the tibial bone defect was devoid of any implanted hydrogel, resulting in a radiolucent gap with no signs of the drill hole being filled. The presence of nHAp in the Zn-CS/nHAp/β-Gp hydrogel resulted in better wound closure and bone formation, in contrast with the findings for hydrogel without nHAp and the control. The radiographs depicted minimal periosteal reaction and smoothening of cortical bone defect edges in the control and Zn-CS/β-GP-treated groups. The shape of the bone defect changed to an oval morphology, suggesting the initiation of bone healing. Histological analysis involving hematoxylin and eosin (H&E) staining (Figure 8b) and collagen staining (Figure 8c) was performed. Initiation of bone formation was found to be more prominent (purple color) in Zn-CS/nHAp/β-GP hydrogel-treated animals, whereas few areas with purple color were observed in Zn-CS/β-Gp hydrogel-treated animals. No apparent bone formation was observed in control animals (Figure 8b). The presence of nHAp in the hydrogel was a key factor in bone crystal deposition and acted as a nucleating site for bone formation. Collagen is the main component of the ECM involved in the bone repair mechanism. It is clear that collagen deposition (blue-colored deposits) was much more intense in Zn-CS/nHAp/β-GP hydrogel-treated animals than in the control or Zn-CS/β-GP hydrogel-treated animals (Figure 8c). Thus, the addition of nHAp in the Zn-CS/β-GP hydrogel improved bone formation and closure of rat tibial bone defects in vivo.

## Conclusions

The current investigations implicate the importance of incorporating nHAp in a thermosensitive CS-based hydrogel to improve its physical and biological characteristics. Increased protein adsorption, controlled swelling, and decreased susceptibility to lysozyme degradation were exhibited with the addition of nHAp. The hydrogel was also found to be osteoconductive in nature, promoting the differentiation of mMSCs into osteoblasts through the upregulation of Runx2 expression. Moreover, the use of Zn-CS/nHAp/β-GP hydrogels promoted bone healing in critical-sized rat tibial defects. The current findings demonstrate the advantages of adding nHAp to hydrogels, implying their potential clinical application in bone regeneration.

## Methods

### Preparation of Zn-doped CS

Zn-doped CS was prepared under refluxing conditions by using an acidic solution of chitosan and a precursor solution for zinc. The procedure has been previously described in detail [48].

### Preparation of Zn-CS/nHAp/β-GP

Zn-doped (2% w/v) CS was weighed and dissolved in 0.1 M acetic acid to obtain a clear solution. β-glycerophosphate (β-GP) disodium salt hydrate (Sigma Aldrich) solutions of appropriate molar concentration were prepared by dissolving calculated amounts of β-GP in distilled water. Both solutions were pre-cooled to 4°C before hydrogel preparation. nHAp (Sigma Aldrich) was sonicated in distilled water at 0.1% (w/v) to obtain a homogeneous dispersion and pre-cooled to 4°C prior to hydrogel formation. To the solution containing Zn-CS/nHAp, β-GP solution was added drop-wise under continuous mixing. The pre-cooled solutions were mixed together at a ratio of 8:1:1 (Zn-CS:β-GP:nHAp) with the final concentration of β-GP in the sol being 0.35 M. The sol form of the hydrogel was poured into 24-well culture plates and incubated at 37°C to allow gelation to take place. Gelation was observed within 10 min at pH 7. The hydrogel was then stored at −20°C overnight, followed by lyophilization at −40°C for 16 h.

### Characterization of the hydrogels

The external surface morphology and porous structure of the synthesized Zn-doped CS and the freeze-dried Zn-CS/nHAp/β-GP hydrogel were analyzed by SEM. Thin sections of scaffolds were sliced using a scalpel and the samples were fixed on adhesive carbon tapes, gold-coated, and subjected to SEM analysis using the HR SEM Quanta 200FEG Instrument (Netherlands). The freeze-dried hydrogel samples were ground into a powder form in liquid nitrogen. The powdered scaffolds were analyzed by XRD with a 2θ angle of 5°–80° at a speed of 2° min^−1^ using an analytical XPERT PRO powder diffractometer operating at a voltage of 40 kV (Cu Kα radiation). The intermolecular chemical interactions between the various functional groups within the components present in the Zn-CS/β-GP hydrogel and the Zn-CS/nHAp/β-GP hydrogel were analyzed by FTIR (American Perkin Elmer Spectrum). The samples were scanned in the range from 4,000 to 450 cm^−1^.

### Swelling studies

Equal amounts of freeze-dried Zn-CS/β-GP and Zn-CS/nHAp/β-GP hydrogels were measured and their dry weights were noted as W_i_. They were then immersed in 1 × PBS for different time periods (1, 6, 12, and 24 h) at 37°C. At the end of each incubation period, the hydrogels were washed with deionized water to remove any ions adsorbed onto the surfaces and blot-dried using filter paper. The wet weights were recorded as W_f_. The swelling ratio of the hydrogel was calculated using the following formula:

$$ {\text{Swelling ratio}} = \left( {{\text{W}}_{\text{i}} {-}{\text{W}}_{\text{t}} } \right)/\left( {{\text{W}}_{\text{i}} } \right) $$

### Biodegradation studies

Equal amounts of hydrogel samples were weighed and their initial weights were noted as W_o_. They were then immersed in medium containing lysozymes (Sigma Aldrich) at a concentration similar to that in circulating levels of blood (10,000 U/L) and incubated for 48 h at 37°C [49]. At the end of the incubation period, the supernatant was removed and the hydrogels were freeze-dried. The final weight of the freeze-dried hydrogels was noted as W_t_. The degradation percentage of the hydrogels was calculated using the following formula:

$$ {\text{Biodegradation }} \% = ({\text{W}}_{\text{o}} {-}{\text{W}}_{\text{t}} )/{\text{W}}_{\text{t}} \times 100 $$

### Protein adsorption studies

Equal amounts of hydrogels were weighed and were subjected to pre-wetting in 100% ethanol for 1 h, followed by incubation in PBS for 30 min to reach equilibrium hydration. They were then incubated from 1 to 24 h in bovine serum albumin solutions (BSA, 5 mg/mL) (Sigma Aldrich) at 37°C. After incubation, the hydrogels were removed and the supernatant solution was used for quantification of unabsorbed proteins by Bradford’s assay.

### Exogenous biomineralization

Equal amount of hydrogels were weighed and tested for their biomineralization abilities by immersing them in simulated body fluid (SBF) for a period of 7 , 14 , and 21 days at 37°C. Equal volumes of fresh SBF were replaced once every 2 days. The SBF solution was prepared according to a previous report [50]. After the desired time intervals, the scaffolds were removed, washed thrice with deionized water, and lyophilized. The freeze-dried samples were then subjected to FTIR and XRD analyses.

### Antimicrobial activity

Antibacterial activity was determined by the zone of inhibition test using *S. pyogenes* (Gram-positive bacteria) and *E. coli* (Gram-negative bacteria). Overnight cultures were prepared. A 100-µL aliquot of the overnight culture of the bacteria (1 × 10^8^ CFU/mL) was spread onto Luria–Bertani agar plates, and 20 mg of the lyophilized hydrogel was placed on the plates, followed by incubation for 24 h at 37°C. The clear area (zone of inhibition) was measured and the results were tabulated.

### Cytotoxicity assessment

Indirect MTT assay was carried out to test the cytotoxicity of the hydrogels. mMSCs (4 × 10^4^ per cm^2^) were seeded in 24-well plates. The freeze-dried Zn-CS/nHAp/β-GP hydrogel (800 mg) was weighed and soaked in 16 mL of DMEM for 24 h. The supernatant, termed as “conditioned medium”, was taken and added at different volumes (25, 50, 100, 200, 300, and 400 µL) to the wells containing cells. Triton X-100 (0.1%) served as a positive control. Cells seeded into the wells with normal medium and incubated for 24 h served as negative controls. The medium was carefully aspirated from the wells, and 200 µL of 0.05% MTT solution was added to each well and incubated for another hour at 37°C. DMSO was used to dissolve the formazan crystals formed, and the optical densities (OD) were measured using a spectrophotometer (ULTRA SPEC 2100 pro; Amersham Life Sciences, USA) at 570 nm.

### Cell morphology evaluation

Zn-CS/β-GP and Zn-CS/β-GP/nHAp hydrogels were UV-treated for 2 h. Approximately 2 × 10^5^ mMSCs were seeded onto the hydrogels and incubated for 4 days. The medium was changed once every 2 days. After 4 days of incubation, the medium was removed and the cells were washed with ice-cold 1 × PBS. Ten milligrams of fluorescein diacetate (FDA) (Sigma Aldrich) was weighed and dissolved in 10 mL of acetone, which served as the stock solution. A working solution was prepared freshly by mixing 30 µL of the stock solution with 10 mL of PBS. One milliliter of this working solution was added onto the films in each well and incubated for 15 min at 37°C in the dark [51]. Cells were observed under a fluorescent microscope with 4× and 10× objectives.

### Western blot analysis

mMSCs were grown in normal or osteogenic medium with Zn-CS/β-GP or Zn-CS/β-GP/nHAp hydrogels for different time periods. The cells were then washed with cold phosphate-buffered saline (PBS), and whole cell lysates were prepared. Twenty micrograms of proteins was resolved using 12% sodium dodecyl sulfate–polyacrylamide gel electrophoresis (SDS-PAGE) and transferred to polyvinylidene difluoride (PVDF) membranes by electroblotting. The membranes were blocked with 5% non-fat milk powder (BioRad) and incubated overnight with a primary antibody at 4°C. The membranes were probed with secondary antibodies conjugated with horseradish peroxidase (HRP). Finally, the bands were visualized by adding Super Signal West Dura Extended Duration Substrate (Thermo Scientific) according to the manufacturer’s instructions. The images obtained were used for quantification with the ImageJ software, as described previously [52]. Mouse monoclonal Runx2 (1:1,000) antibodies, Cdk2 (1:1,000) antibodies, and secondary antibodies conjugated with HRP were obtained from Santa Cruz Biotechnology. Cdk2 antibodies served as internal loading controls.

### Real-time RT-PCR analysis

mMSCs were grown for different time periods in normal or osteogenic medium conditioned with Zn-CS/β-GP or Zn-CS/β-GP/nHAp hydrogels. Total RNA was isolated using the Trizol method (Invitrogen) according to the manufacturer’s instructions. cDNA synthesis was carried out with 1 μg of total RNA from each sample using the GeNei AMV RT-PCR kit according to the manufacturer’s protocol. SYBR reagent (BioRad) was used for real-time RT-PCR with mouse-specific oligonucleotide primers, as shown in Table 2. Relative mRNA expression was calculated by using the ΔΔCt method of relative quantification [34].

**Table 2:** Primer sequences used for real-time RT-PCR analysis (F: forward; R: reverse)

Gene	Primer sequence (forward/reverse)
Runx2	5ʹ CGCCTCACAAACAACCACAG 3ʹ (F) 5ʹ TCACTGTGCTGAAGAGGCTG 3ʹ (R)
ALP	5ʹ TTGTGCCAGAGAAAGAGAGAGA 3ʹ (F) 5ʹ GTTTCAGGGCATTTTTCAAGGT 3ʹ (R)
COL-I	5ʹ TAACCCCCTCCCCAGCCACAAA 3ʹ (F) 5ʹ TTCCTCTTGGCCGTGCGTCA 3ʹ (R)
OC	5ʹ ATGGCTTGAAGACCGCCTAC 3ʹ (F) 5ʹ AGGGCAGAGAGAGAGGACAG 3ʹ (R)
GAPDH	5ʹ GAGAGACCCCACTTGCTGCCA 3′(F) 5′ CTCACACTGCCCCTCCCTGGT 3′ (R)

### In vivo examination of bone formation in drill hole defects in rat tibia

Male Wistar rats (3 months old) weighing 200–250 g were kept in a room with light (12 h light–dark cycle) at a controlled temperature. The procedure for creating drill hole defects was approved by the animal ethical committee, Kovai Medical College and Hospital, Coimbatore, India. Rats were divided into three groups (n = 6 in each group): (1) control, (2) Zn-CS/β-GP, and (3) Zn-CS/β-GP/nHAp. The rats were anesthetized with 10% ketamine and 2% xylazine (1:1, 0.1 mL/100 g body weight, i.m.) and subjected to perforation of the right tibia by using a dental drill (diameter, 3 mm) under constant saline irrigation (0.9% NaCl). The defects were entirely filled with each of the hydrogels in the respective experimental groups. The defects in control animals were left unfilled. After 14 days, the animals were sacrificed under anesthesia and the tibiae were removed and radiographed. The tibiae were then fixed in neutral 10% buffered formalin for 48 h at room temperature. The specimens were embedded in paraffin after decalcification, sectioned at a thickness of 6 μm, and examined with H&E staining and collagen (Masson’s trichrome) staining.

### Statistical analysis

All experiments were performed in triplicates and the results have been expressed as the mean ± standard deviation (S.D.) Statistical significance was calculated by ANOVA and Student’s *t* test analyses. A *p* value of lower than 0.05 was considered to be statistically significant.

## Abbreviations

3D: three-dimensional
Zn: zinc
CS: chitosan
nHAp: nanohydroxyapatite
β-GP: beta-glycerophosphate
SEM: scanning electron microscopy
EDX: energy dispersive spectroscopy
FTIR: Fourier transform infrared spectroscopy
XRD: X-ray diffraction
MTT: (3-(4,5-dimethylthiazol-2-yl)-2,5-diphenyltetrazolium bromide)
RT-PCR: reverse transcriptase polymerase chain reaction
ECM: extracellular matrix
GAGs: glycosaminoglycans
ALP: alkaline phosphatase
hBMSCs: human bone marrow stem cells
JCPDS: joint committee on powder diffraction standards
SBF: simulated body fluid
mMSCs: mouse mesenchymal stem cells
COL-1: type-I collagen
OC: osteocalcin
FDA: fluorescein diacetate
SDS-PAGE: sodium dodecyl sulfate–polyacrylamide gel electrophoresis
PVDF: polyvinylidene fluoride
HRP: horseradish peroxidase
